# *Giardia duodenalis* infection in the context of a community-based deworming and water, sanitation and hygiene trial in Timor-Leste

**DOI:** 10.1186/s13071-019-3752-9

**Published:** 2019-10-18

**Authors:** Jessica Y. H. Aw, Naomi E. Clarke, James S. McCarthy, Rebecca J. Traub, Salvador Amaral, Md Hamidul Huque, Ross M. Andrews, Darren J. Gray, Archie C. A. Clements, Susana Vaz Nery

**Affiliations:** 1 Research School of Population Health, Australian National University, Canberra, ACT Australia; 2 The Kirby Institute for Infection and Immunity in Society, University of New South Wales, Sydney, NSW Australia; 3 Clinical Tropical Medicine Laboratory, QIMR Berghofer Medical Research Institute, Brisbane, QLD Australia; 4 Faculty of Veterinary and Agricultural Sciences, University of Melbourne, Parkville, VIC Australia; 5 Menzies School of Health Research, Charles Darwin University, Darwin, NT Australia; 6 Faculty of Health Sciences, Curtin University, Perth, WA Australia

**Keywords:** *Giardia duodenalis*, Giardiasis, Deworming, Albendazole, Water, Sanitation, Hygiene

## Abstract

**Background:**

Giardiasis is a common diarrhoeal disease caused by the protozoan *Giardia duodenalis*. It is prevalent in low-income countries in the context of inadequate access to water, sanitation and hygiene (WASH), and is frequently co-endemic with neglected tropical diseases such as soil-transmitted helminth (STH) infections. Large-scale periodic deworming programmes are often implemented in these settings; however, there is limited evidence for the impact of regular anthelminthic treatment on *G. duodenalis* infection. Additionally, few studies have examined the impact of WASH interventions on *G. duodenalis*.

**Methods:**

The WASH for WORMS cluster randomised controlled trial was conducted in remote communities in Manufahi municipality, Timor-Leste, between 2012 and 2016. All study communities received four rounds of deworming with albendazole at six-monthly intervals. Half were randomised to additionally receive a community-level WASH intervention following study baseline. We measured *G. duodenalis* infection in study participants every six months for two years, immediately prior to deworming, as a pre-specified secondary outcome of the trial. WASH access and behaviours were measured using questionnaires.

**Results:**

There was no significant change in *G. duodenalis* prevalence in either study arm between baseline and the final study follow-up. We found no additional benefit of the community-level WASH intervention on *G. duodenalis* infection (relative risk: 1.05, 95% CI: 0.72–1.54). Risk factors for *G. duodenalis* infection included living in a household with a child under five years of age (adjusted odds ratio, aOR: 1.35, 95% CI: 1.04–1.75), living in a household with more than six people (aOR: 1.32, 95% CI: 1.02–1.72), and sampling during the rainy season (aOR: 1.23, 95% CI: 1.04–1.45). Individuals infected with the hookworm *Necator americanus* were less likely to have *G. duodenalis* infection (aOR: 0.71, 95% CI: 0.57–0.88).

**Conclusions:**

Prevalence of *G. duodenalis* was not affected by a community WASH intervention or by two years of regular deworming with albendazole. Direct household contacts appear to play a dominant role in driving transmission. We found evidence of antagonistic effects between *G. duodenalis* and hookworm infection, which warrants further investigation in the context of global deworming efforts.

*Trial registration* Australian New Zealand Clinical Trials Registry, ACTRN12614000680662. Registered 27 June 2014, retrospectively registered. https://anzctr.org.au/Trial/Registration/TrialReview.aspx?id=366540.
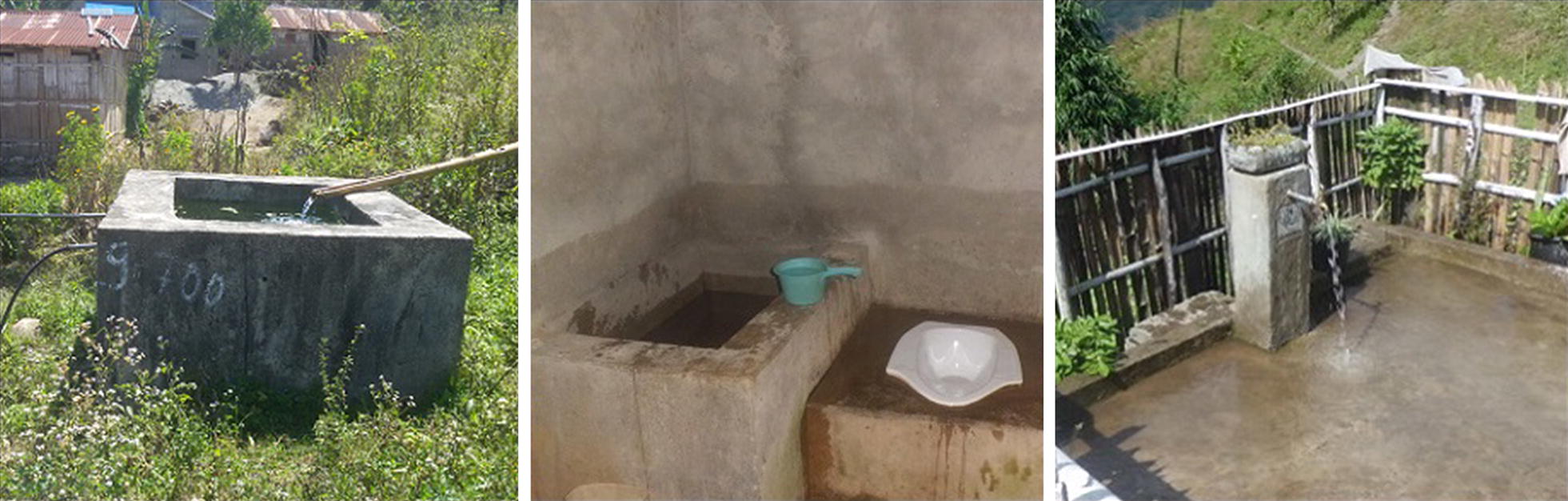

## Background

Giardiasis is a parasitic enteric disease caused by the protozoan *Giardia duodenalis*. It is one of the most frequent enteric protozoan infections worldwide, with a global disease burden of approximately 280 million cases annually. *Giardia duodenalis* is most prevalent in low-resource settings [1], where prevalence between 20 and 40% is common [2–8], with prevalences approaching 70% also reported [9–11]. The health impact of giardiasis ranges from asymptomatic carriage, to acute self-limiting diarrhoeal disease, to chronic gastrointestinal illness accompanied by malabsorption [1]. Chronic giardiasis in childhood has been associated with poor growth, malnutrition, and decreased cognitive function [10, 12–15]. Giardiasis is transmitted by the faecal-oral route, directly from person to person, or indirectly through contaminated water or food [1]. Zoonotic transmission is also possible, with dogs and cats posing greatest risk of transmitting *Giardia* to humans [16–19].

*Giardia duodenalis* is frequently co-endemic with other parasitic diseases that are common in low-resource settings, of which soil-transmitted helminth (STH) infection is one of the most prevalent [20, 21]. STHs are parasitic intestinal helminths, including roundworm (*Ascaris lumbricoides*), hookworms (*Necator americanus*, *Ancylostoma duodenale* and *Ancylostoma ceylanicum*), whipworm (*Trichuris trichiura*), and threadworm (*Strongyloides stercoralis*). While *G. duodenalis* control relies on treatment with antimicrobial agents following diagnosis, control of STH infections in endemic settings occurs mainly through large-scale mass deworming programmes, as recommended by the World Health Organization (WHO) [22]. In the context of a global commitment to controlling STH infections and other neglected tropical diseases, more than 700 million children were treated with the anthelminthic agents albendazole or mebendazole in deworming campaigns in 2017 [23].

There is limited literature regarding the impact of regular deworming on *G. duodenalis* infection. Albendazole is efficacious in treating giardiasis when repeated doses are given over several days [24]; however, reported efficacy of single dose albendazole, as used in deworming programmes, is variable [25–27]. Several cross-sectional studies in areas receiving regular deworming have noted ongoing high prevalence of *G. duodenalis* [3, 8], and a small randomised controlled trial (RCT) in Bangladesh described an increase in *G. duodenalis* infection in the context of regular deworming with mebendazole [28, 29]. Some studies have shown that STH infections are associated with a decreased risk of *G. duodenalis* infection [30], while others have conversely noted an increased risk of protozoan infections among those infected with STHs [9, 31].

Given transmission of giardiasis by the faecal-oral route and the role of environmental contamination, interventions aimed at improving water, sanitation and hygiene (WASH) may play a role in reducing *G. duodenalis* transmission. A systematic review and meta-analysis of mainly observational studies found that latrine access, latrine use, and treating household drinking water were all associated with lower odds of *G. duodenalis* infection [32]. The effect of WASH interventions on *G. duodenalis* infection has been examined in small number of intervention studies, with mixed findings. In a longitudinal study in Ethiopia, a significant reduction in *G. duodenalis* prevalence was observed following a school-based WASH intervention [33], while in several small RCTs, household water treatment interventions showed no effect on *Giardia* infections [34–36]. An RCT conducted in the context of the Indian Total Sanitation Campaign reported a slightly lower *Giardia* prevalence among children who lived in villages where a community-based sanitation intervention was implemented [37]. In the recent WASH Benefits RCTs, conducted in Kenya and Bangladesh, the impact of WASH and nutrition interventions on child growth, diarrhoea, and enteric infections was studied. In Bangladesh, a significantly lower prevalence of *G. duodenalis* was found following sanitation, handwashing, and combined WASH interventions [5]. However, in Kenya, no interventions reduced *G. duodenalis* prevalence [6]. In the Sanitation Hygiene Infant Nutrition Efficacy (SHINE) trial, conducted in rural Zimbabwe, a household-level WASH intervention had no impact on the prevalence of giardiasis among infants in the first 12 months of life, though there was evidence of reduced prevalence following a combined WASH and nutrition intervention [38].

The WASH for WORMS cluster RCT was conducted in Timor-Leste to investigate the impact of a community WASH and deworming intervention, compared to deworming alone, on intestinal parasite infections. The impact of the study intervention on the primary outcome (STH infections) has been published separately [39]. Prevalence of the protozoan infections *G. duodenalis*, *Entamoeba histolytica* and *Cryptosporidum* spp. were pre-specified secondary outcomes of the trial [40]. Baseline data indicated that *G. duodenalis* infection was relatively common, with an overall prevalence of 13%, while *E. histolytica* and *Cryptosporidium* spp. were present in less than 0.1% of the study population [41].

In this paper, we aimed to determine the impact of a community-based WASH intervention on *G. duodenalis* infection; examine the prevalence of *G. duodenalis* over time in the context of regular deworming; and identify risk factors for *G. duodenalis* infection.

## Methods

### Study design

**Fig. 1:**
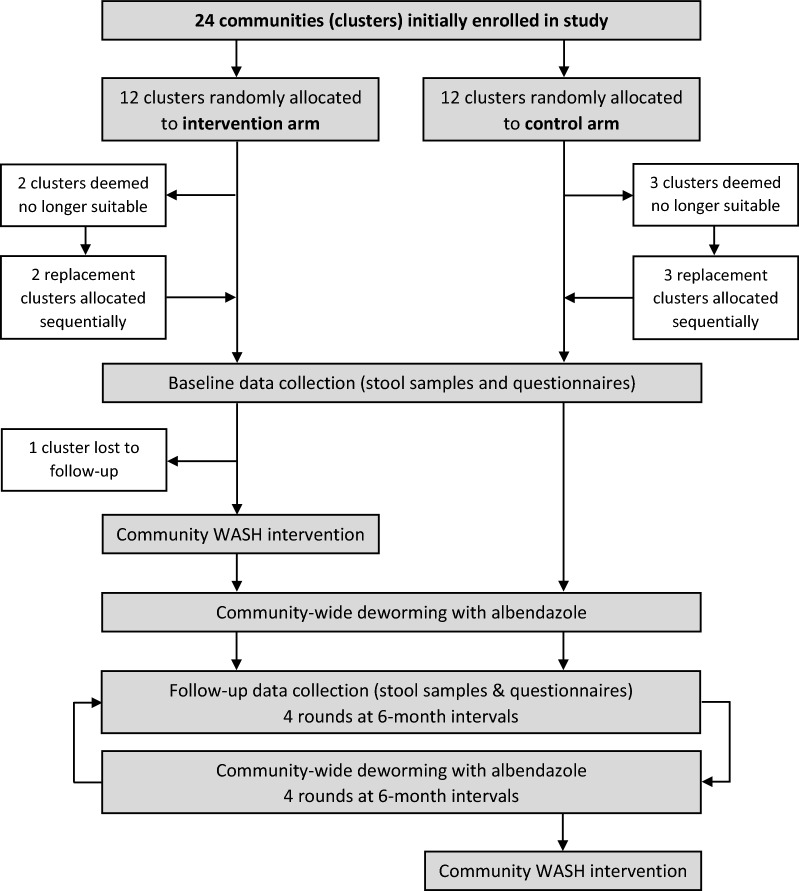
Flow diagram of the WASH for WORMS trial

WASH for WORMS was a two-arm cluster RCT conducted between 2012 and 2016 in the Manufahi municipality of Timor-Leste [40]. Communities in the control arm received regular community-wide deworming, while communities in the intervention arm additionally received an integrated community-based WASH intervention. Informed by initial sample size requirements based on the primary outcomes (hookworm and *Ascaris* spp. infections), 24 communities were enrolled in the study and randomly allocated to a study arm. Five communities (two intervention and three control) were deemed ineligible prior to trial commencement and were replaced sequentially, rather than randomly, from a pre-generated list of replacement communities. It was not feasible to implement randomisation for the additional communities due to factors related to implementation of the WASH intervention by the partner organisation, including timing and community expectations. One community in the intervention arm was subsequently lost to follow-up after study baseline. Therefore, a total of 23 communities completed the study, and 18 communities (nine intervention and nine control) followed the randomisation protocol [40]. A flow diagram of the study is shown in Fig. 1.

### Study intervention

The deworming intervention, delivered in both study arms, involved distributing a single 400 mg dose of albendazole to all eligible community members (those older than one year of age, excluding pregnant women in the first trimester). Children aged 12–23 months were given half a dose. Four deworming rounds, conducted at six-monthly intervals, were delivered, with a fifth round given following completion of data collection. Albendazole was delivered by members of the research team and taken under direct observation.

The WASH intervention, implemented following study baseline and prior to the first deworming round, consisted of providing access to a protected community water supply, promoting improved sanitation through encouraging household latrine construction [42], and conducting hygiene education, focused on handwashing before eating and after defecating. The WASH intervention was implemented by WaterAid Australia, an international non-governmental organisation (NGO) in conjunction with local partner NGOs. Further details regarding the WASH and deworming interventions are available in the published study protocol [40].

### Data collection

Sociodemographic data (age, sex, education, employment, household income and assets), clinical information (current and recent diarrhoea), and WASH information (handwashing behaviours, shoe-wearing, defecation practices, presence and type of household latrine, household garbage disposal, and household water supply) were collected through individual and household-level questionnaires at baseline and four follow-up time points, at six-monthly intervals. Stool samples were collected from all willing community members over one year of age, at baseline and each of the four follow-up time points, immediately prior to deworming. Samples were preserved in 5% potassium dichromate and sent to the QIMR Berghofer Medical Research Institute (Brisbane, Australia) for analysis. DNA extractions were performed using the PowerSoil DNA Isolation Kit (Mo Bio, Carlsbad, CA, USA) and subjected to two multiplex real-time PCR reactions: one for detection of *G. duodenalis*, *Cryptosporidium* spp., *Entamoeba histolytica* and *Strongyloides* spp., and the other for detection of *Ascaris* spp., *N. americanus*, *Ancylostoma* spp. and *Trichuris* spp. An Equine Herpes Virus target was spiked into each sample as an extraction and internal qPCR control [43].

### Statistical analysis

Prevalence of *G. duodenalis* infection along with 95% confidence intervals (CI) was calculated for each study arm at each time point using logistic regression models that accounted for community-level clustering using robust standard errors. The impact of the community-level WASH intervention on *G. duodenalis* infection was evaluated in the 18 communities that followed the randomisation protocol, across all four follow-up time points. We constructed a generalised linear mixed model, with community, household, and individual entered as random effects, to account for repeated observations on the same individuals over time, nested within households and communities. We used Poisson regression to model relative risk (RR), incorporating an interaction term between study arm and follow-up time point, and adjusting for age group and sex. We calculated RR (and 95% CI) of infection for the study intervention, compared to the control arm, at each follow-up time point, by calculating a post-estimation linear combination of coefficients and standard errors, using Wald-type methods [39].

To explore individual and household-level risk factors for *G. duodenalis* infection, we conducted an observational analysis of all individuals in the study cohort (i.e. in the 23 communities that completed the study), across all five study time points (baseline and four follow-ups). We again constructed generalised linear mixed models to account for clustering at the community, household, and individual level. We used Bernoulli logistic regression to model odds ratio (OR) for each predictor variable. Predictor variables included sociodemographic factors, WASH access and behaviours, household animal ownership, STH infections and season. Socioeconomic status was determined using principal component analysis as described previously [41, 44]. A full list of variables examined is provided in Additional file 1: Text S1. Univariable analyses were conducted for each potential risk factor, with variables retained if *P *< 0.2. Multivariable models were then constructed using a two-stage approach. “Within-domain” multivariable models were built for groups of related variables: demographic, individual socioeconomic, individual hygiene, individual sanitation, school sanitation, household socioeconomic, household hygiene, household water, household sanitation, environmental, and infection-related (see Additional file 1: Text S1). These models were built using variables retained from the univariable analysis, adjusted for age, sex, and study time point. A full model was then constructed with variables significant at *P *< 0.1 in the “within-domain” models, adjusted for age, sex and study time point. The final multivariable model was produced using backward stepwise regression such that age, sex, study time point, and covariates significant at *P *< 0.05 remained.

Finally, we examined the impact of previous *G. duodenalis* infections on risk of infection in the entire study cohort. For this model, the outcome variable was *G. duodenalis* infection at the final study time point, and the predictor variable was the number of infections at previous study time points, adjusted for age and sex, and clustering at the community and household levels. All statistical analyses were conducted using Stata version 15 (College Station, TX, USA).

## Results

At study baseline, among the 23 communities that completed the trial, 2448 participants provided informed consent to participate in the study. Of these, 1971 (80.5%) provided a stool sample that was analysed for *G. duodenalis* infection. Baseline characteristics of this study population are shown in Additional file 2: Table S1. Participation rates across the course of the study, including those for the subset of 18 communities that followed the randomisation protocol, are summarised in Additional file 2: Table S2. Treatment coverage of eligible community members with albendazole was high, above 90% at each study time point [39].

### *Giardia duodenalis* infections over time

Prevalence of *G. duodenalis* infection across the study period in each arm, overall and disaggregated by age, is shown in Table 1. Intensity of infection, measured as qPCR cycle threshold (Cq) values, across the study period is depicted in Additional file 2: Table S3. At baseline, *G. duodenalis* prevalence in the intervention arm was 14.5% (95% CI: 10.1–17.6%) and in the control arm was 12.3% (95% CI: 9.6–16.3%). At the end of the study, *G. duodenalis* prevalence in the intervention and control arms was 17.4% (95% CI: 12.9–21.6%) and 14.0% (95% CI: 10.3–18.0%), respectively. As depicted in Table 1, in both study arms, *G. duodenalis* prevalence was highest among children aged 1–5 years of age, ranging between 23.7% (95% CI: 14.4–30.1%) and 34.3% (95% CI: 25.2–43.4%) in the intervention arm, and between 14.4% (95% CI: 5.5–23.1%) and 38.1% (95% CI: 27.7–48.5%) in the control arm. There was no significant difference in risk of *G. duodenalis* infection at the end of the study compared to baseline, in either the intervention arm (RR: 1.17, 95% CI: 0.87–1.86, *P *= 0.222) or the control arm (RR: 1.16, 95% CI: 0.90–1.48, *P *= 0.252).

**Table 1:** Prevalence of *G. duodenalis* in the study population over time

	Overall		1–5 years		6–11 years		12–17 years		18–64 years		65+ years	
	*n*	Prevalence (95% CI)	*N*	Prevalence (95% CI)	*N*	Prevalence (95% CI)	*N*	Prevalence (95% CI)	*N*	Prevalence (95% CI)	*n*	Prevalence (95% CI)
Baseline												
Intervention	710	14.5 (10.1–17.6)	131	23.7 (14.4–30.1)	157	25.5 (18.6–32.4)	65	13.9 (4.4–22.8)	306	6.9 (3.0–10.1)	51	3.9 (0–8.7)
Control	888	12.3 (9.6–16.3)	127	29.9 (20.9–39.6)	175	19.4 (13.4–25.4)	118	12.7 (5.7–21.0)	395	5.1 (2.4–8.5)	73	4.1 (0–9.2)
Follow-up 1												
Intervention	584	15.4 (8.8–22.3)	128	30.5 (18.2–42.0)	133	24.1 (15.1–32.0)	44	11.4 (1.0–22.6)	240	5.4 (1.8–8.9)	39	2.6 (0–7.5)
Control	689	10.2 (4.7–14.2)	104	14.4 (5.5–23.1)	151	20.5 (11.2–26.9)	72	9.7 (1.6–16.9)	290	5.5 (2.1–8.9)	72	1.4 (0–4.1)
Follow-up 2												
Intervention	552	16.5 (11.8–20.8)	113	26.5 (17.9–35.0)	134	19.4 (12.7–26.1)	51	9.8 (1.6–18.0)	210	12.9 (6.5–18.3)	44	6.8 (0–14.6)
Control	624	11.2 (7.7–14.8)	98	25.5 (16.5–34.6)	147	12.9 (7.5–18.4)	62	19.4 (9.5–29.2)	253	4.3 (1.2–7.2)	64	4.7 (0–9.9)
Follow-up 3												
Intervention	531	19.6 (12.5–24.4)	119	32.8 (18.9–42.3)	123	26.0 (18.3–33.8)	44	22.7 (9.8–36.2)	194	10.8 (4.1–16.3)	51	3.9 (0–9.2)
Control	609	11.7 (7.2–16.0)	92	28.3 (16.1–40.6)	131	16.8 (10.4–23.2)	66	10.6 (2.0–18.5)	256	5.9 (2.0–1.06)	64	1.6 (0–4.6)
Follow-up 4												
Intervention	553	17.4 (12.9–21.6)	105	34.3 (25.2–43.4)	136	26.5 (19.1–33.9)	68	14.7 (6.3–23.1)	197	6.6 (3.1–10.1)	47	2.1 (0–7.0)
Control	623	14.0 (10.3–18.0)	84	38.1 (27.7–48.5)	135	19.3 (12.6–25.9)	65	16.9 (7.8–26.0)	261	5.4 (2.6–8.1)	78	5.1 (0–11.0)

*Note*: The table includes participants in the 18 communities that were randomly allocated

*Abbreviation*: CI, confidence interval

Across all age groups and study time points, the prevalence of self-reported diarrhoea (at the time of survey or within the previous 2 weeks) among those infected with *G. duodenalis* was 11.5% (95% CI: 7.4–17.5%). This was not significantly different between the intervention arm (14.1%, 95% CI: 7.7–24.5%) and the control arm (8.6%, 95% CI: 5.4–13.7%, *P *= 0.206), and was also not significantly different to the prevalence of diarrhoea reported among those not infected with *G. duodenalis* (11.5%, 95% CI: 9.7–13.7%, *P *= 0.768). Among those infected with *G. duodenalis*, the highest diarrhoea prevalence was observed in children aged 1–5 years in both the intervention arm (15.9%, 95% CI: 8.5–28.0%) and the control arm (11.9%, 95% CI: 6.5–20.6%).

### Impact of WASH intervention

The results of the generalised linear mixed model examining the impact of the study intervention are shown in Table 2. These results demonstrate no significant difference in risk of *G. duodenalis* infection between the intervention or control arm at any time point. At the completion of the trial, the RR of *G. duodenalis* infection in the intervention arm, compared to the control arm, was 1.05 (95% CI: 0.72–1.54, *P *= 0.787).

**Table 2:** Impact of the study intervention on *G. duodenalis* infection

Study time point	Study arm	*n*	Infection prevalence		
			Prevalence (95% CI)	Adjusted RR^a^ (95% CI)	*P*-value
Follow-up 1	Intervention	584	15.4 (8.8–22.3)	1.28 (0.77–2.14)	0.341
	Control	689	10.2 (4.7–14.2)		
Follow-up 2	Intervention	552	16.5 (11.8–20.8)	1.26 (0.83–1.94)	0.277
	Control	624	11.2 (7.7–14.8)		
Follow-up 3	Intervention	531	19.6 (12.5–24.4)	1.38 (0.91–2.10)	0.124
	Control	609	11.7 (7.2–16.0)		
Follow-up 4	Intervention	553	17.4 (12.9–21.6)	1.05 (0.72–1.54)	0.787
	Control	623	14.0 (10.3–18.0)		

*Notes:* ^a^Adjusted RR obtained from generalised linear mixed model, adjusted for age group and sex (fixed effects) and clustering at the community, household, and individual levels (random effects). The model included 1878 participants in 456 households in 18 clusters (those randomly allocated to intervention and control arms)

*Abbreviations:* CI, confidence interval; RR, relative risk

### Risk factors for *G. duodenalis* infection

Univariable analyses found various WASH, demographic and socioeconomic variables significantly associated with *G. duodenalis* infection at *P *< 0.2; full results of univariable analyses are provided in Additional file 2: Table S4. In the final adjusted multivariable model (Table 3), the odds of *G. duodenalis* infection decreased significantly with increasing age. People who lived in a household with a child under five years of age had significantly increased odds of infection compared to those who did not (adjusted odds ratio, aOR: 1.35, 95% CI: 1.04–1.75, *P* = 0.023). Similarly, those living in a household of more than six people had higher odds of infection compared to those living in households of six or fewer people (aOR: 1.32, 95% CI: 1.02–1.72, *P* = 0.035). Odds of infection were higher in the wet season compared to the dry season (aOR: 1.23, 95% CI: 1.04–1.45, *P* = 0.015). Infection with *Necator americanus* was associated with lower odds of *G. duodenalis* infection (aOR: 0.71, 95% CI: 0.57–0.88, *P *= 0.002). No WASH variables remained as significant predictors of infection in the final model.

**Table 3:** Results of final generalised linear mixed model of risk factors for *G. duodenalis* infection

Covariate	aOR	95% CI	*P-*value
Age group (years)^a^			
6–11	**0.74**	**0.57–0.95**	**0.017**
12–17	**0.42**	**0.30–0.60**	**< 0.001**
18–64	**0.13**	**0.10–0.78**	**< 0.001**
65+	**0.10**	**0.06–0.16**	**< 0.001**
Male sex	0.97	0.80–1.19	0.802
Study time point^b^			
Follow-up 1	**0.77**	**0.60–0.98**	**0.037**
Follow-up 2	0.88	0.68–1.13	0.318
Follow-up 3	0.97	0.75–1.25	0.820
Follow-up 4	1.05	0.81–1.37	0.689
Lives in a household with at least one child under 5 years old	**1.35**	**1.04–1.75**	**0.023**
Lives in a household with more than 6 people	**1.32**	**1.02–1.72**	**0.035**
Infection with *Necator americanus*	**0.71**	**0.57–0.88**	**0.002**
Wet season (December through May)^c^	**1.23**	**1.04–1.45**	**0.015**
Random effects variance (95% CI)			
Community	0.26 (0.11**–**0.65)		
Household	1.20 (0.88**–**1.63)		
Participant	1.20 (0.88**–**1.65)		

*Notes*: Results in bold text are those significant at *P *< 0.05. Reference groups are as follows: ^a^age 1**–**5 years; ^b^study baseline; ^c^dry season (June through November). The model includes 2694 people in 604 households in 23 communities

*Abbreviations*: aOR, adjusted odds ratio; CI, confidence interval

### Repeated infections in individuals over time

Table 4 shows the impact of *G. duodenalis* infections at previous study time points on the risk of infection with *G. duodenalis* at the final follow-up. The risk of *G. duodenalis* infection at the end of the trial was significantly greater among those with a history of infection diagnosed at previous study time points, with relative risk of infection increasing with the number of time points.

**Table 4:** Impact of previous infections on *G. duodenalis* infection at the end of the trial

	*n*	Prevalence at final follow-up (95% CI)	Relative risk	95% CI	*P-*value
No infection at previous time points	1082	8.5 (7.0–10.3)	reference		
Infection at one previous time point	254	26.3 (21.3–32.1)	**2.05**	**1.47–2.86**	**< 0.001**
Infection at two previous time points	90	53.3 (43.0–63.3)	**3.81**	**2.62–5.56**	**< 0.001**
Infection at three previous time points	26	53.8 (35.0–71.6)	**3.88**	**2.13–7.07**	**< 0.001**
Infection at four previous time points	12	66.7 (37.6–86.9)	**5.55**	**2.55–12.10**	**< 0.001**

*Notes*: Effect estimates obtained using generalised linear mixed models, adjusted for age group and sex (fixed effects) and clustering at the community and household levels (random effects). The model included 1464 individuals in 438 households in 23 communities. Results in bold text are those significant at *P *< 0.05

*Abbreviation*: CI, confidence interval

## Discussion

To our knowledge, this is the first study to examine *G. duodenalis* infection in the context of a community-based WASH and deworming RCT. In our study setting in rural Timor-Leste, we observed a moderate overall prevalence of *G. duodenalis* in both study arms, ranging between 10.2 and 19.6% over the study period. Prevalence was highest (up to 38.1%) among children aged 1**–**5 years. This is consistent with the known epidemiology of *Giardia* infection that tends to predominate among young children [1, 45, 46]. Most infections appeared to be subclinical; only 11.5% of individuals infected with *G. duodenalis* reported diarrhoea, which was similar to the background diarrhoea prevalence among the study population. Over four rounds of community-wide deworming with albendazole, we observed no significant change in *G. duodenalis* prevalence in either study arm. This supports previous findings that a single 400 mg dose of albendazole is not efficacious against *G. duodenalis* [25].

We found no impact of the community-based WASH intervention in terms of reducing *G. duodenalis* prevalence, compared to the control arm that did not receive a WASH intervention. This is consistent with the findings of the primary outcome of the WASH for WORMS trial, where no additional impact of the WASH intervention on STH infections was identified [39]. Our findings are also consistent with several previous intervention studies, including the recent SHINE trial and WASH Benefits Kenya, that identified no impact of household-level WASH interventions on *Giardia* infection [6, 38]. However, other studies, including WASH Benefits Bangladesh, have detected lower risk of *G. duodenalis* infection among participants who received WASH interventions [5, 37]. A possible explanation for our findings is suboptimal coverage of the WASH intervention. As previously reported, although initial household latrine coverage was high following the WASH intervention, this decreased over time, and at the end of the trial, 40% participants in the intervention arm were practising open defecation [39]. This would facilitate environmental contamination, likely leading to ongoing transmission of *G. duodenalis*. Additionally, although rates of reported handwashing with soap were high among study participants at the end of the trial, this was self-reported and may therefore overestimate true handwashing behaviours [47]. Other potential sources of ongoing *G. duodenalis* transmission following the WASH intervention include zoonotic transmission and contaminated drinking water. Given that protected water sources were provided as part of the study intervention, contamination occurring at the main source of community drinking water is unlikely. However, it is possible that individuals obtained drinking water from other (unprotected) sources, or that water contamination occurred during or after collection. WASH interventions may require higher coverage, over a longer duration of time, to achieve any impact on transmission of intestinal protozoa in areas with significant environmental contamination.

Our risk factor analysis provides further insight into the individual-level factors associated with *G. duodenalis* infection. We examined a wide range of factors including WASH access and behaviours, animal ownership, STH infections, and socioeconomic variables. However, relatively few predictors remained in our final model. Age group was a major predictor of infection, and *N. americanus* infection was associated with reduced odds of *G. duodenalis* infection. This potential antagonism between hookworm and *G. duodenalis* has been identified previously [30], with potential explanations including competitive inhibition within the small intestine, or cross-immunity due to helminth-induced Th2 cell response [30]. However, these findings are not consistent across the literature, with other studies reporting increased risk of protozoan infections among those infected with STH [9, 31].

Other factors that significantly affected the odds of *G. duodenalis* infection included living in a household with a child under five years of age, and living in a household with more than six people. This demonstrates that direct household contacts likely play a dominant role in transmission. Additionally, infection was significantly more likely during the wet season, consistent with previous findings of increased transmission during rainy seasons [11, 48].

We identified that previous infection with *G. duodenalis* was associated with significantly increased odds of infection at the end of the study, with a dose-response observed with increasing number of previous infections. Although there is some evidence that people may develop a degree of acquired immunity to *G. duodenalis* [49], it has also been shown that reinfections occur commonly and rapidly after successful treatment [50]. Furthermore, it is known that giardiasis can become chronic and persist for more than six months following initial infection [14, 51]. Therefore, repeated infections within individuals in this study could represent either chronic infections, or reinfections occurring after clearing *Giardia*, potentially driven by persistent environmental exposure or repeated risk behaviours.

This analysis was conducted in the context of a robustly-designed RCT, using a highly-sensitive diagnostic technique; however, there are several important limitations. The community-based WASH intervention, although delivered in accordance with national sanitation policy by local NGOs in a culturally-appropriate manner, did not achieve its aims of 100% household latrine coverage and “open-defecation free” communities. This made detecting any impact of the WASH intervention less likely. As mentioned above, data relating to WASH access and behaviours were derived predominantly from self-report, which could be biased towards reporting safer WASH behaviours [47], leading to difficulty in detecting true associations between WASH behaviours and infection. Finally, despite including a wide range of potential predictors in the analysis of risk factors for *G. duodenalis* infection, residual confounding due to unmeasured factors remains likely.

A number of key priorities for ongoing research arise from this study. First, additional research examining the impact of WASH risk factors and WASH interventions on protozoan infections is required. Most previous studies on risk factors have focused on children [32]. Given that we identified infection across all age groups and that many households are intergenerational, more research into *G. duodenalis* infection across the entire community should be undertaken. Identifying improved mechanisms to measure WASH behaviours in a research context is also required, to allow accurate determination of key risk factors and inform the optimal design of interventions. Food-borne transmission is an important source of *G. duodenalis* infection [52, 53] and *Giardia* also has potential for zoonotic transmission [17–19]; therefore, WASH interventions may be improved by incorporating education about safe food handling practice, and hygiene around animals, tailored to local contexts.

Additional studies are required to investigate the potential antagonistic relationship between *G. duodenalis* and hookworm infections, and to identify ways to mitigate this potentially antagonistic effect. This is particularly important given the high burden of both STH and *Giardia* infections in low-resource settings, especially among children, and the large-scale global deworming efforts currently underway. It may be necessary for STH control guidelines to include recommendations for undertaking diagnosis and treatment of *G. duodenalis* infections during monitoring efforts, and for educating communities about the symptoms of giardiasis and the importance of seeking additional treatment given that deworming is unlikely to be effective. Strengthening the understanding of the role of both WASH and deworming in the epidemiology and control of *G. duodenalis* is crucial, in order to inform the design and delivery of interventions tailored to the communities that will benefit from them most.

## Conclusions

This study demonstrates the ongoing burden of *G. duodenalis* across all age groups, but particularly among children, in communities receiving regular deworming for STH control. It also demonstrates some of the challenges involved in implementing and evaluating WASH interventions in low-income settings, and the complexities in generating evidence to demonstrate the impact of WASH on health outcomes. This study provides important evidence to inform the understanding of *G. duodenalis* epidemiology in the context of regular deworming and WASH interventions, and highlights important areas for future research, including further investigation of a potentially antagonistic relationship between hookworm and *G. duodenalis* infections.

## Supplementary information

**Additional file 1: Text S1.** Full list of variables examined as risk factors for *Giardia* infection.

**Additional file 2: Table S1.** Baseline characteristics of study participants. **Table S2.** Participation rates in the study over time. **Table S3.** Intensity of infection with *G. duodenalis* over time, measured using qPCR cycle threshold (Cq) values among positive samples. **Table S4.** Results of univariable analyses for risk factors for *G. duodenalis* infection (*n *= 2333), based on generalised linear mixed models accounting for village, household and individual-level clustering.

## Data Availability

Data are available from the corresponding author on request.

## Abbreviations

aOR: adjusted odds ratio
CI: confidence interval
Cq: cycle threshold
NGO: non-governmental organisation
OR: odds ratio
qPCR: quantitative polymerase chain reaction
RCT: randomised controlled trial
RR: relative risk
STH: soil-transmitted helminth
WASH: water, sanitation and hygiene
WHO: World Health Organization

## References

[1] Huang DB, White AC (2006). An updated review on *Cryptosporidium* and *Giardia*. Gastroenterol Clin North Am..

[2] Caron Y, Hong R, Gauthier L, Laillou A, Wieringa F, Berger J (2018). Stunting, beyond acute diarrhoea: *Giardia duodenalis*, in Cambodia. Nutrients..

[3] Quihui-Cota L, Morales-Figueroa GG (2012). Persistence of intestinal parasitic infections during the national de-worming campaign in schoolchildren of northwestern Mexico: a cross-sectional study. Ann Gastroenterol..

[4] Farthing MJ, Mata L, Urrutia JJ, Kronmal RA (1986). Natural history of *Giardia* infection of infants and children in rural Guatemala and its impact on physical growth. Am J Clin Nutr..

[5] Lin A, Ercumen A, Benjamin-Chung J, Arnold BF, Das S, Haque R (2018). Effects of water, sanitation, handwashing, and nutritional interventions on child enteric protozoan infections in rural Bangladesh: a cluster-randomized controlled trial. Clin Infect Dis..

[6] Pickering AJ, Njenga SM, Steinbaum L, Swarthout J, Lin A, Arnold BF (2019). Effects of single and integrated water, sanitation, handwashing, and nutrition interventions on child soil-transmitted helminth and *Giardia* infections: a cluster-randomized controlled trial in rural Kenya. PLoS Med..

[7] Esrey SA, Collett J, Miliotis MD, Koornhof HJ, Makhale P (1989). The risk of infection from *Giardia lamblia* due to drinking water supply, use of water, and latrines among preschool children in rural Lesotho. Int J Epidemiol..

[8] Ferreira FS, Baptista-Fernandes T, Oliveira D, Rodrigues R, Neves E, Lima A (2015). *Giardia duodenalis* and soil-transmitted helminths infections in children in São Tomé and Príncipe: do we think *Giardia* when addressing parasite control?. J Trop Pediatr..

[9] Chard AN, Baker KK, Tsai K, Levy K, Sistrunk JR, Chang HH (2019). Associations between soil-transmitted helminthiasis and viral, bacterial, and protozoal enteroinfections: a cross-sectional study in rural Laos. Parasit Vectors..

[10] Ignatius R, Gahutu JB, Klotz C, Steininger C, Shyirambere C, Lyng M (2012). High prevalence of *Giardia duodenalis* Assemblage B infection and association with underweight in Rwandan children. PLoS Negl Trop Dis..

[11] Siwila J, Phiri IG, Enemark HL, Nchito M, Olsen A (2011). Seasonal prevalence and incidence of *Cryptosporidium* spp and *Giardia duodenalis* and associated diarrhoea in children attending pre-school in Kafue, Zambia. Trans R Soc Trop Med Hyg..

[12] Al-Mekhlafi MH, Azlin M, Nor Aini U, Shaik A, Sa’iah A, Fatmah M (2005). Giardiasis as a predictor of childhood malnutrition in Orang Asli children in Malaysia. Trans R Soc Trop Med Hyg..

[13] Berkman DS, Lescano AG, Gilman RH, Lopez SL, Black MM (2002). Effects of stunting, diarrhoeal disease, and parasitic infection during infancy on cognition in late childhood: a follow-up study. Lancet..

[14] Halliez MC, Buret AG (2013). Extra-intestinal and long term consequences of *Giardia duodenalis* infections. World J Gastroenterol..

[15] Rogawski ET, Bartelt LA, Platts-Mills JA, Seidman JC, Samie A, Havt A (2017). Determinants and impact of *Giardia* infection in the first 2 years of life in the MAL-ED birth cohort. J Pediatric Infect Dis Soc..

[16] Bouzid M, Halai K, Jeffreys D, Hunter PR (2015). The prevalence of *Giardia* infection in dogs and cats, a systematic review and meta-analysis of prevalence studies from stool samples. Vet Parasitol..

[17] Traub R, Monis P, Robertson I, Irwin P, Mencke N, Thompson R (2004). Epidemiological and molecular evidence supports the zoonotic transmission of *Giardia* among humans and dogs living in the same community. Parasitology..

[18] Thompson RA (2004). The zoonotic significance and molecular epidemiology of *Giardia* and giardiasis. Vet Parasitol..

[19] Traub RJ, Inpankaew T, Reid SA, Sutthikornchai C, Sukthana Y, Robertson ID (2009). Transmission cycles of *Giardia duodenalis* in dogs and humans in Temple communities in Bangkok - a critical evaluation of its prevalence using three diagnostic tests in the field in the absence of a gold standard. Acta Trop..

[20] Steinmann P, Utzinger J, Du Z-W, Zhou X-N (2010). Multiparasitism: a neglected reality on global, regional and local scale. Adv Parasitol..

[21] Dacal E, Saugar JM, de Lucio A, Hernández-de-Mingo M, Robinson E, Köster PC (2018). Prevalence and molecular characterization of *Strongyloides stercoralis*, *Giardia duodenalis*, *Cryptosporidium* spp., and *Blastocystis* spp. isolates in school children in Cubal. Western Angola. Parasit Vectors..

[22] WHO (2017). Preventive chemotherapy to control soil-transmitted helminth infections in at-risk population groups.

[23] WHO (2018). Schistosomiasis and soil-transmitted helminthiases: numbers of people treated in 2017. Wkly Epidemiol Rec..

[24] Solaymani-Mohammadi S, Genkinger JM, Loffredo CA, Singer SM (2010). A meta-analysis of the effectiveness of albendazole compared with metronidazole as treatments for infections with *Giardia duodenalis*. PLoS Negl Trop Dis..

[25] Pungpak S, Singhasivanon V, Bunnag D, Radomyos B, Nibaddhasopon P, Harinasuta K (1996). Albendazole as a treatment for *Giardia* infection. Ann Trop Med Parasitol..

[26] Hall A, Nahar Q (1993). Albendazole as a treatment for infections with *Giardia duodenalis* in children in Bangladesh. Trans R Soc Trop Med Hyg..

[27] Speich B, Marti H, Ame SM, Ali SM, Bogoch II, Utzinger J (2013). Prevalence of intestinal protozoa infection among school-aged children on Pemba Island, Tanzania, and effect of single-dose albendazole, nitazoxanide and albendazole-nitazoxanide. Parasit Vectors..

[28] Rousham E (1994). An increase in *Giardia duodenalis* infection among children receiving periodic anthelmintic treatment in Bangladesh. J Trop Pediatr..

[29] Northrop-Clewes CA, Rousham EK, Mascie-Taylor CN, Lunn PG (2001). Anthelmintic treatment of rural Bangladeshi children: effect on host physiology, growth, and biochemical status. Am J Clin Nutr..

[30] Blackwell AD, Martin M, Kaplan H, Gurven M (2013). Antagonism between two intestinal parasites in humans: the importance of co-infection for infection risk and recovery dynamics. Proc Biol Sci..

[31] Hagel I, Cabrera M, Puccio F, Santaella C, Buvat E, Infante B (2011). Co-infection with *Ascaris lumbricoides* modulates protective immune responses against *Giardia duodenalis* in school Venezuelan rural children. Acta Trop..

[32] Speich B, Croll D, Fürst T, Utzinger J, Keiser J (2016). Effect of sanitation and water treatment on intestinal protozoa infection: a systematic review and meta-analysis. Lancet Infect Dis..

[33] Gelaye B, Kumie A, Aboset N, Berhane Y, Williams MA (2014). School-based intervention: evaluating the role of water, latrines and hygiene education on trachoma and intestinal parasitic infections in Ethiopia. J Water Sanit Hyg Dev..

[34] Hellard ME, Sinclair MI, Forbes AB, Fairley CK (2001). A randomized, blinded, controlled trial investigating the gastrointestinal health effects of drinking water quality. Environ Health Perspect..

[35] Crump JA, Mendoza CE, Priest JW, Glass RI, Monroe SS, Dauphin LA (2007). Comparing serologic response against enteric pathogens with reported diarrhea to assess the impact of improved household drinking water quality. Am J Trop Med Hyg..

[36] Zambrano LD, Priest JW, Ivan E, Rusine J, Nagel C, Kirby M (2017). Use of serologic responses against enteropathogens to assess the impact of a point-of-use water filter: a randomized controlled trial in Western Province, Rwanda. Am J Trop Med Hyg..

[37] Patil SR, Arnold BF, Salvatore AL, Briceno B, Ganguly S, Colford JM (2014). The effect of India’s total sanitation campaign on defecation behaviors and child health in rural Madhya Pradesh: a cluster randomized controlled trial. PLoS Med..

[38] Rogawski McQuade ET, Platts-Mills JA, Gratz J, Zhang J, Moulton LH, Mutasa K (2019). Impact of water quality, sanitation, handwashing, and nutritional interventions on enteric infections in rural Zimbabwe: the Sanitation Hygiene Infant Nutrition Efficacy (SHINE) Trial. J Infect Dis..

[39] Vaz Nery S, Traub RJ, McCarthy JS, Clarke NE, Amaral S, Weking E (2019). WASH for WORMS: a cluster-randomized controlled trial of the impact of a community-integrated water, sanitation, and hygiene and deworming intervention on soil-transmitted helminth infections. Am J Trop Med Hyg..

[40] Nery SV, McCarthy JS, Traub R, Andrews RM, Black J, Gray D (2015). A cluster-randomised controlled trial integrating a community-based water, sanitation and hygiene programme, with mass distribution of albendazole to reduce intestinal parasites in Timor-Leste: the WASH for WORMS research protocol. BMJ Open..

[41] Campbell SJ, Nery SV, D’Este CA, Gray DJ, McCarthy JS, Traub RJ (2016). Water, sanitation and hygiene related risk factors for soil-transmitted helminth and *Giardia duodenalis* infections in rural communities in Timor-Leste. Int J Parasitol..

[42] Kar K, Chambers R (2008). Handbook on community-led total sanitation.

[43] Llewellyn S, Inpankaew T, Nery S, Gray D, Verweij J, Clements A (2016). Application of a multiplex quantitative PCR to assess prevalence and intensity of intestinal parasite infections in a controlled clinical trial. PLoS Negl Trop Dis..

[44] Vaz Nery S, Clarke NE, Richardson A, Traub R, McCarthy JS, Gray DJ (2019). Risk factors for infection with soil-transmitted helminths during an integrated community level water, sanitation, and hygiene and deworming intervention in Timor-Leste. Int J Parasitol..

[45] Choy SH, Al-Mekhlafi HM, Mahdy MA, Nasr NN, Sulaiman M, Lim YA (2014). Prevalence and associated risk factors of *Giardia* infection among indigenous communities in rural Malaysia. Sci Rep..

[46] Reynoldson J, Behnke J, Gracey M, Horton R, Spargo R, Hopkins R (1998). Efficacy of albendazole against *Giardia* and hookworm in a remote Aboriginal community in the north of Western Australia. Acta Trop..

[47] Manun’Ebo M, Cousens S, Haggerty P, Kalengaie M, Ashworth A, Kirkwood B (1997). Measuring hygiene practices: a comparison of questionnaires with direct observations in rural Zaïre. Trop Med Int Health..

[48] Ayalew D, Boelee E, Endeshaw T, Petros B (2008). *Cryptosporidium* and *Giardia* infection and drinking water sources among children in Lege Dini, Ethiopia. Trop Med Int Health..

[49] Solaymani-Mohammadi S, Singer SM (2010). *Giardia duodenalis*: the double-edged sword of immune responses in giardiasis. Exp Parasitol..

[50] Gilman RH, Marquis GS, Miranda E, Vestegui M, Martinez H (1988). Rapid reinfection by *Giardia lamblia* after treatment in a hyperendemic Third World community. Lancet..

[51] Robertson LJ, Hanevik K, Escobedo AA, Mørch K, Langeland N (2010). Giardiasis - why do the symptoms sometimes never stop?. Trends Parasitol..

[52] Kirk MD, Pires SM, Black RE, Caipo M, Crump JA, Devleesschauwer B (2015). World Health Organization estimates of the global and regional disease burden of 22 foodborne bacterial, protozoal, and viral diseases, 2010: a data synthesis. PLoS Med..

[53] Mohammed Mahdy A, Lim Y, Surin J, Wan KL, Al-Mekhlafi MH (2008). Risk factors for endemic giardiasis: highlighting the possible association of contaminated water and food. Trans R Soc Trop Med Hyg..

