# Supplementary material for: Giardia duodenalis infection in the context of a community-based deworming and water, sanitation and hygiene trial in Timor-Leste
Source: Parasit Vectors. 2019 Oct 18;12:491. doi: 10.1186/s13071-019-3752-9 (PMC6798381; doi:10.1186/s13071-019-3752-9)
Supplement: Supplementary file 1 — Additional file 1: Text S1. Full list of variables examined as risk factors for Giardia infection. [file 13071_2019_3752_MOESM1_ESM.pdf]

Additional file 1: Text S1. Full list of variables examined as risk factors for *Giardia duodenalis* infection Unless otherwise specified, variables are binary

Demographic variables

- Age group (*categorical: 1-5 years; 6-11 years; 12-17 years; 18-64 years; 65+ years*)
- Sex
- Study follow-up number (*categorical: baseline; follow-up 1; follow-up 2; follow-up 3; follow-up 4*)

Individual hygiene variables

- Washes hands using soap or ash
- Washes hands before contact with food
- Washes hands after contact with faeces
- Washes hands after contact with dirt
- Always wears shoes indoors
- Always wears shoes outdoors and while toileting

Individual sanitation variables

- Main place of defecation is toilet
- Practises open defecation
- Uses water to clean self after defecation

School sanitation variables (if aged 6-17 years)

- Uses toilet at school

Individual socioeconomic variables (if aged 18+ years)

- Education level (*categorical: never went to school; not finished primary; finished primary but not secondary; finished secondary or higher*)
- Employment (*categorical: no employment outside the home; employed as farmer; employed as other*)

Household sanitation variables

- Household has toilet
- Household toilet has slab
- Household toilet is pour-flush latrine
- Household toilet observed to be clean
- Water available to clean self after defecating
- Household toilet is shared with another household
- Child waste disposed of hygienically
- Household garbage disposed of in bush
- Household garbage disposed of by digging/burying
- Household garbage disposed of by burning

Household water variables

- Household main water source (*categorical: piped water; protected spring; unprotected spring/dugwell; tubewell/borehole; surface water*)
- Distance to water source is more than 15 minutes
- Water always available from main water source
- All household water stored in covered containers
- Household water treated before drinking

Household hygiene variables

- Household has a food garden
- Household main food preparer washes hands before preparing food
- Number of dogs owned (*categorical: none; 1 or 2; 3 or more*)
- Number of pigs owned (*categorical: none; 1 or 2; 3 or more*)
- Number of chickens owned (*categorical: none; 1 to 5; 6 or more*)
- Households owns cow(s)
- Household owns horse(s)

Household socioeconomic variables

- At least one child under 5 years of age in household
- More than 6 people living in dwelling
- Socioeconomic quintile (*categorical: quintile 1 poorest; quintile 2; quintile 3; quintile 4; quintile 5 richest*)

Environmental variables

- Season (*wet/dry*)

Infection-related variables

- *Ascaris* spp. infection
- *N. americanus* infection
- *Ancylostoma* spp. infection
- *Trichuris* spp. infection
