# Supplementary material for: Giardia duodenalis infection in the context of a community-based deworming and water, sanitation and hygiene trial in Timor-Leste
Source: Parasit Vectors. 2019 Oct 18;12:491. doi: 10.1186/s13071-019-3752-9 (PMC6798381; doi:10.1186/s13071-019-3752-9)
Supplement: Supplementary file 2 — Additional file 2: Table S1. Baseline characteristics of study participants. Table S2. Participation rates in the study over time. Table S3. Intensity of infection with G. duodenalis over time, measured using qPCR cycle threshold (Cq) values among positive samples. Table S4. Results of univariable analyses for risk factors for G. duodenalis infection (n = 2333), based on generalised linear mixed models accounting for village, household and individual-level clustering. [file 13071_2019_3752_MOESM2_ESM.pdf]

Additional file 2: Table S1. Baseline characteristics of study participants*

	**Intervention arm**	**Control arm**
**Individual variables**		
Demographics and clinical information	*n=1103*	*n=1290*
Female	581 (52.7%)	638 (49.5%)
Mean (SD) age in years	26.0 (21.6)	26.8 (21.7)
5 years and under	202 (18.3%)	189 (14.7%)
Between 5 and 18 years	346 (31.4%)	441 (34.3%)
18 years and over	554 (50.3%)	657 (51.0%)
Current or recent diarrhea†	149 (14.2%)	99 (7.8%)
For children aged 6-17	*n=325*	*n=439*
Attends school	293 (90.2%)	366 (83.4%)
For adults over 18 years	*n=548*	*n=641*
Has never been to school	213 (38.9%)	286 (44.6%)
Employed‡	454 (82.9%)	572 (89.2%)
Protozoan and STH infections	*n=893*	*n=1078*
G. duodenalis infections	120 (13.4%)	139 (12.9%)
Ascaris spp. infections	233 (26.1%)	204 (18.9%)
N. americanus infections	536 (60.0%)	635 (58.9%)
Ancylostoma spp. infections	46 (5.2%)	54 (5.0%)
T. trichiura infections	6 (0.7%)	2 (0.2%)
S. stercoralis infections	1 (0.1%)	0
**Household variables**	*n=263*	*n=302*
Has household toilet	53 (20.2%)	71 (23.5%)
Main water source is unprotected	200 (76.1%)	248 (82.1%)
Household income < $1USD/day	132 (50.2%)	144 (47.7%)
Owns a motor vehicle	22 (8.4%)	32 (10.6%)
Has electricity	152 (57.8%)	254 (84.1%)

* Study participants are defined as residents who were present at the time of the baseline visit and provided questionnaires or stool samples. Participants from all 23 communities that completed the study are included in this table. † Defined as participants who had self-reported diarrhea at the time of questionnaire, or within the previous 2 weeks. ‡ Being employed includes all work done outside the house.

Table S2. Participation rates in the study over time

	Baseline		Follow-up 1		Follow-up 2		Follow-up 3		Follow-up 4	
	**Intervention**	**Control**	**Intervention**	**Control**	**Intervention**	**Control**	**Control**	**Intervention**	**Control**	**Intervention**
**All 23 communities (11 intervention, 12 control) that completed the trial**										
Households (n)	286	320	288	323	288	324	288	324	288	324
Residents present (n)	1199	1365	1083	1225	1025	1110	1033	1138	993	1134
Provided informed consent, n (%)*	1129 (94.2%)	1319 (96.6%)	1023 (94.5%)	1170 (95.5%)	934 (91.1%)	1062 (95.7%)	912 (88.2%)	1010 (88.8%)	891 (89.7%)	1010 (89.1%)
Provided stool, n (%)†	893 (79.1%)	1078 (81.7%)	735 (71.8%)	887 (75.8%)	698 (74.7%)	796 (75.0%)	688 (75.4%)	803 (79.5%)	681 (76.4%)	783 (77.5%)
Provided both stool and questionnaire, n (%)†	855 (75.7%)	1055 (80.0%)	722 (70.6%)	876 (74.8%)	678 (72.6%)	780 (73.4%)	664 (72.8%)	801 (79.3%)	652 (73.2%)	760 (75.3%)
**18 communities (9 intervention, 9 control) that followed the randomisation protocol**										
Households (n)	241	252	244	253	244	254	244	254	244	254
Residents present (n)	991	1109	907	975	869	880	866	879	843	909
Provided informed consent, n (%)*	929	1073	840	907	761	848	732	758	730	794
Provided stool, n (%)†	711 (76.5%)	891 (83.0%)	584 (69.5%)	689 (76.0%)	552 (72.5%)	624 (73.6%)	531 (72.5%)	609 (80.3%)	553 (75.7%)	623 (78.5%)
Provided both stool and questionnaire, n (%)†	673 (72.4%)	871 (81.2%)	573 (68.2%)	681 (75.1%)	534 (70.2%)	612 (70.2%)	507 (69.3%)	607 (80.1%)	525 (71.9%)	618 (77.8%)

* Denominator is all residents present. † Denominator is all residents present, over 1 year of age, who provided informed consent.

Table S3. Intensity of infection with *G. duodenalis* over time, measured using qPCR cycle threshold (Cq) values[*] among positive samples

	Overall		1–5 years		6–11 years		12-17 years		18-64 years		65+ years	
	Number positive samples	Mean (SD) Cq value	Number positive samples	Mean (SD) Cq value	Number positive samples	Mean (SD) Cq value	Number positive samples	Mean (SD) Cq value	Number positive samples	Mean (SD) Cq value	Number positive samples	Mean (SD) Cq value
**Baseline**												
Intervention	104	23.5 (3.7)	31	24.7 (3.4)	40	23.4 (3.9)	9	22.2 (2.7)	21	23.0 (4.4)	2	22.2 (1.7)
Control	110	24.3 (3.5)	38	25.1 (3.4)	34	24.0 (3.6)	15	24.4 (3.5)	20	23.1 (3.3)	3	25.5 (1.1)
**Follow-up 1**												
Intervention	90	24.7 (3.6)	39	24.9 (3.4)	32	24.7 (4.1)	5	25.4 (4.0)	13	23.7 (3.0)	1	19.6 (NA)
Control	70	24.0 (3.2)	15	23.7 (2.6)	31	25.4 (3.0)	7	21.1 (2.7)	16	22.8 (2.9)	1	24.8 (NA)
**Follow-up 2**												
Intervention	91	23.7 (3.0)	30	24.5 (3.5)	26	23.6 (3.1)	5	23.2 (2.5)	27	23.0 (2.6)	3	23.2 (0.8)
Control	70	25.0 (3.7)	25	24.9 (3.8)	19	24.8 (3.1)	12	24.2 (3.8)	11	25.3 (3.8)	3	29.6 (4.3)
**Follow-up 3**												
Intervention	104	25.3 (4.1)	39	25.8 (4.3)	32	24.6 (3.7)	10	24.1 (3.0)	21	26.4 (4.9)	2	22.4 (1.8)
Control	71	24.6 (3.9)	26	25.0 (4.2)	22	23.8 (3.2)	7	24.8 (4.9)	15	25.2 (4.0)	1	23.0 (NA)
**Follow-up 4**												
Intervention	96	26.2 (3.9)	36	26.9 (4.0)	36	25.3 (4.0)	10	25.5 (3.8)	13	27.5 (3.5)	1	25.3 (NA)
Control	87	25.6 (4.0)	32	25.9 (4.0)	26	25.2 (4.2)	11	26.3 (4.9)	14	24.3 (3.0)	4	27.8 (3.2)

SD = standard deviation; NA = not applicable. [*] The cycle threshold (Cq) value represents the number of PCR cycles required for the fluorescence signal of the amplified DNA products to cross a threshold value that exceeds background level. Higher quantities of DNA, reflecting more intense infections, therefore result in lower Cq values. Table includes participants in the 18 communities that were randomly allocated.

Table S4. Results of univariable analyses for risk factors for *G. duodenalis* infection (N=2333), based on generalised linear mixed effects models accounting for village, household and individual-level clustering

Covariate	Odds ratio	95% CI	*p* value
General variables			
**Age group**[a]			
6–11 years	**0.68**	**0.53–0.87**	**0.002**
12–17 years	**0.38**	**0.27–0.53**	**<0.001**
18–64 years	**0.12**	**0.09–0.16**	**<0.001**
65+ years	**0.08**	**0.05–0.13**	**<0.001**
Male sex	1.05	0.84–1.30	0.661
**Study follow-up time point**[b]			
Follow-up 1	0.86	0.67–1.09	0.218
Follow-up 2	1.03	0.81–1.31	0.793
Follow-up 3	1.09	0.86–1.39	0.483
Follow-up 4	**1.23**	**0.97–1.57**	**0.086**
Individual hygiene variables			
Washes hands using soap or ash	**1.21**	**0.97–1.52**	**0.092**
Washes hands before contact with food	**0.71**	**0.59–0.85**	**<0.001**
Washes hands after contact with faeces	1.13	0.92–1.38	0.236
Washes hands after contact with dirt	**0.73**	**0.61–0.87**	**<0.001**
Always wears shoes indoors	**0.86**	**0.71–1.05**	**0.139**
Always wears shoes outdoors and while toileting	**0.64**	**0.53–0.76**	**<0.001**
Individual sanitation variables			
Main place of defecation is toilet	0.91	0.74–1.13	0.399
Practises open defecation	**1.26**	**1.03–1.55**	**0.027**
Uses water to clean self after defecation	0.98	0.80–1.20	0.820
School sanitation variables (children age 6–17 years only)			
Uses toilet at school	0.80	0.56–1.14	0.224
Individual socioeconomic variables			
Education level *(adults age 18+ years only)*[c]			
Didn't finish primary school	**1.51**	**0.86–2.63**	**0.149**
Finished primary but not secondary school	**1.86**	**1.10–3.16**	**0.021**
Finished secondary school or higher	1.53	0.75–3.10	0.243
Employment *(adults age 18+ years only)*[d]			
Employed – farmer	0.95	0.62–1.45	0.803
Employed – other job	**0.63**	**0.32–1.22**	**0.170**
Household sanitation variables			
Household has toilet	1.05	0.85–1.30	0.642
Household toilet has slab	0.98	0.72–1.32	0.886
Household toilet is pour-flush latrine	0.97	0.72–1.33	0.866
Household toilet observed to be clean	0.99	0.72–1.35	0.939
Water available to clean self after defecating	0.95	0.68–1.33	0.761
Household toilet is shared with another household	1.17	0.75–1.84	0.481
Child waste disposed of hygienically	**1.37**	**0.96–1.96**	**0.084**
Household garbage disposed of in bush	**1.16**	**0.96–1.39**	**0.131**
Household garbage disposed of by digging/burying	0.85	0.61–1.18	0.335
Household garbage disposed of by burning	0.95	0.80–1.13	0.573

Household water variables			
Household main water source[e]			
Tubewell/borehole	**0.93**	**0.59–1.46**	**0.743**
Unprotected spring/dugwell	0.95	0.76–1.18	0.625
Protected spring	1.11	0.57–2.19	0.757
**Surface water**	**0.74**	**0.54–1.02**	**0.062**
Distance to water source is more than 15 minutes	0.95	0.77–1.18	0.655
Water always available from main water source	1.00	0.79–1.26	0.985
Household water stored in only covered containers	0.87	0.63–1.19	0.380
Household water treated	1.01	0.84–1.22	0.884
Household hygiene variables			
Household has a food garden	**0.73**	**0.20–1.06**	**0.096**
Household main food preparer washes hands before preparing food	1.02	0.82–1.28	0.829
Number of dogs owned by household[f]			
1 or 2	1.23	0.84–1.81	0.283
3 or more	1.28	0.86–1.91	0.218
Number of pigs owned by household[f]			
1 or 2	0.85	0.61–1.19	0.340
3 or more	0.95	0.67–1.36	0.786
Number of chickens owned by household[f]			
1 to 5	0.94	0.65–1.35	0.718
6 or more	1.05	0.72–1.54	0.798
Household owns cow(s)	0.97	0.78–1.22	0.815
Household owns horse(s)	0.93	0.75–1.16	0.530
Household socioeconomic variables			
At least one child under 5 years of age in household	**2.21**	**1.72–2.84**	**<0.001**
More than 6 people living in dwelling	**1.73**	**1.33–2.25**	**<0.001**
Socioeconomic quintile[g]			
Quintile 2	0.83	0.68–1.26	0.636
Quintile 3	0.99	0.71–1.37	0.939
Quintile 4	0.94	0.66–1.33	0.715
Quintile 5 (richest)	1.05	0.73–1.52	0.785
Environmental variables			
Wet season (December through May)[h]	**1.26**	**1.07–1.48**	**0.005**
Infection-related variables			
Ascaris spp. infection	1.14	0.89–1.47	0.309
N. americanus infection	**0.55**	**0.45–0.68**	**<0.001**
Ancylostoma spp. infection	0.69	0.35–1.33	0.267
Trichuris spp. infection	0.49	0.129–1.87	0.297

Results in bold: Covariates entered in multivariable regression models (p<0.2 in univariable analyses). Reference categories are as follows: [a] Age 1–5 years, [b] Study baseline, [c] Never went to school, [d] No employment outside the home, [e] Household water source is piped water, [f] No animals of that type owned, [g] Socioeconomic quintile 1 (poorest), [h] Dry season (June through November).
